# Preoperative evaluation and influencing factors of sentinel lymph node detection for early breast cancer with contrast-enhanced ultrasonography: What matters

**DOI:** 10.1097/MD.0000000000025183

**Published:** 2021-04-02

**Authors:** Shihui Ma, Yuguang Xu, Feihai Ling

**Affiliations:** a Breast Center; b Ultrasound Imaging Department, Zhongshan City People's Hospital, Guangdong Province, China.

**Keywords:** breast cancer, contrast-enhanced ultrasonography, nursing, oncology, sentinel lymph node, treatment

## Abstract

Sentinel lymph node (SLN) is important in the early diagnosis of breast cancer. We aimed to evaluate the role of contrast-enhanced ultrasonography (CEUS) in the preoperative evaluation for SLN and potentially influencing factors, to provide evidence to the management of breast cancer.

Patients with breast cancer who treated in our hospital from May 2018 to May 2020 were selected. All patients underwent CEUS examination to find SLN and judged whether the lymph node had cancer metastasis. We evaluated the sensitivity, specificity, and accuracy of CEUS in predicting SLN, and its differences in pathological diagnosis results and related influencing factors were also analyzed.

A total of 108 patients with breast cancer were included. And a total of 248 SLNs were detected. The sensitivity of CEUS to the preoperative evaluation of SLN was 84.67%, the specificity was 81.14%, the positive predictive value was 76.08%, and the negative predictive value was 89.27%, the positive likelihood ratio was 4.06, and the negative likelihood ratio was 0.14. The area under the curve of the preoperative evaluation of SLN in CEUS examination was 0.813 (95% confidence interval: 0.765–0.911), and there was significant difference in the size of SLNs between SLN-negative and SLN-positive groups (*P* = .043).

Preoperative CEUS has good predictive value for the SLN detection in patients with breast cancer, and it is worthy of clinical application.

## 1 Introduction

Sentinel lymph node (SLN) biopsy is currently a routine procedure for evaluating the axillary staging of early breast cancer,^[1]^ and it's been reported that it can accurately evaluate the pathological status of axillary lymph nodes.^[2,3]^ For patients with negative axillary lymph nodes, SLN biopsy can safely and effectively replace axillary lymph node dissection and improve the quality of life of patients.^[4]^ The commonly used tracing methods of SLN biopsy in clinic are radionuclide method and blue dye method. It's been reported that the combination of those 2 methods can significantly increase the success rate of SLN biopsy and reduce its false negative rate.^[5,6]^ The blue dye method currently uses methylene blue more frequently in China, which is simple, economical, and easy to obtain,^[7]^ but it's been reported to be connected with several adverse complications such as contrast agent extravasation.^[8]^ The radionuclide method recommends the use of 99mTc-labeled sulfur colloid. The manufacturing process of this tracer is relatively complicated with higher expenses, so it has not been widely used.^[9]^ Therefore, it's necessary to identify practical and reliable method for evaluating the SLN.

Ultrasound contrast agent and related technology are important developments in the field of ultrasound medicine in the past decades. Particularly, contrast-enhanced ultrasonography (CEUS) is a new technology that has been continuously developed and improved in recent years.^[10]^ At present, contrast-enhanced ultrasound has been widely used in clinical diagnosis such as the differentiation of benign and malignant tumors in abdominal and superficial organs.^[11]^ There are, however, very few studies on the role of CEUS in identifying and predicting preoperative diagnosis in patients with breast cancer. Therefore, in this study, we aimed to evaluate the effects of CEUS in identifying and predicting preoperative diagnosis in patients with breast cancer, to provide insights into clinical diagnosis and treatment of breast cancer.

## 2 Methods

### 2.1 Ethical consideration

Our study had been verified and approved by the medical ethical commissions of our hospital (No. 20180047-3a), and written informed consents had been obtained from all the included patients.

### 2.2 Patients

We selected patients with breast cancer who were hospitalized in our department from May 2018 to May 2020 as the study population. The criteria for entry of patients were as follows: Preoperative needle biopsy was conducted to confirm the breast cancer by the department of pathology our hospital; The physical examination of 2 experienced breast surgeons in our hospital did not find obvious enlarged lymph nodes. In addition, no suspicious metastatic lymph nodes were found in the routine detection of preoperative color Doppler ultrasonography, and the patients signed and agreed to receive SLN biopsy; patients signed and agreed to use CEUS to evaluate SLN status before operation. The exclusion criteria of this study were patients with pathological diagnosis of inflammatory breast cancer; patients with positive axillary lymph nodes confirmed by puncture pathology; pregnant patients; patients with previous breast surgery or axillary surgery; and patients who did not agree to participant in this study.

### 2.3 CEUS detection

CEUS detection was conducted in comply with related guidelines.^[12,13]^ The patient took the supine position, and the affected upper limb took the external rotation position. After the affected areola area was anesthetized, the doctor took 2 mL of sonovi contrast agent and injected 0.5 mL subcutaneously around the ring areola at 3, 6, 9, and 12 o’clock, and massaged the injection site appropriately, and started the CPS imaging system at the same time. The probe was traced from the enhanced area to the enhanced lymphatic vessels to the axillary area. The first group of enhanced lymph nodes was marked as SLN and marked on the body surface. If the SLN was not visible, the suspicious lymph node was repeatedly explored at the end of the enhanced lymphatic vessel, which was marked as SLN. If the lymphatic vessel was not visualized, the suspicious lymph node was repeatedly explored at a regular location and marked as SLN. In addition, we switched the color Doppler ultrasound to the conventional ultrasound mode and measure the size of the marked SLN.

During breast cancer surgery, all patients underwent tracheal intubation anesthesia. A curved surgical incision was made in the axillary area near the lateral edge of the pectoralis major muscle, the skin, and subcutaneous tissue were cut, and the target lymph node was found along the enhanced lymphatic vessels in the area marked on the ultrasound-contrast body surface. The position and shape of the lymph node were observed, and the size was measured. We compared with the lymph nodes marked by contrast-enhanced ultrasound and confirm that they are the same lymph node. After removal of the lymph node, a quick pathological examination was sent. If the pathological results suggested that the lymph node was not invaded, then axillary lymph node dissection was not performed. If the pathological results suggested that the lymph node was invaded, then we continued the axillary lymph node dissection. The contrast injection in this study was done by the same experienced and skilled breast surgeons.

### 2.4 Materials and diagnosis

Philips X200 color Doppler ultrasound diagnostic apparatus were used, and the probe frequency was 7.15 MHz. Contrast agent used Sulfur hexafluoride microbubbles for injection (SonoMex) was produced by the Geniss company (Italy). The use of ultrasound equipment, the setting of contrast conditions, and the interpretation of ultrasound results were all operated by the same experienced sonographers. Type I is considered as an uninvaded lymph node, and types II and III were all considered as suspected metastatic lymph nodes.^[14]^ The pathological diagnosis of SLN in this study was taken as the criterion standard.

### 2.5 Statistical processing

SPSS 23.00 statistical software was used for analysis. The t test was conducted to compare the differences between the 2 groups, the area under the receiver operating characteristic curve was used to evaluate the diagnostic accuracy of CEUS. *P* < 0.05 indicated that the difference was statistically significant.

## 3 Results

### 3.1 The characteristics of included patients

A total of 108 patients with breast cancer were included, and the characteristics of included patients are presented in Table 1.

**Table 1:** The characteristics of included patients.

Items	Variables
Ages (y)	48.12 ± 3.96
Menopause	39 (36.11%)
Pathological type	
Nonspecific invasive carcinoma	102 (94.44%)
Mucinous carcinoma	4 (3.71%)
Apocrine carcinoma	2 (1.85%)
Pathological grades	
Grade I	19 (17.59%)
Grade II	62 (57.41%)
Grade III	27 (25%)
Estrogen receptor positive	71 (65.74%)
Progesterone receptor positive	71 (65.74%)
Human epidermal growth factor receptor 2 positive	29 (26.85%)
Primary tumor size (cm)	2.13 ± 1.05
SLN size (cm)	1.53 ± 0.48
SLN positive	33 (30.56%)

SLN = sentinel lymph node.

### 3.2 The reference value of CEUS for SLN detection

**Figure 1:**
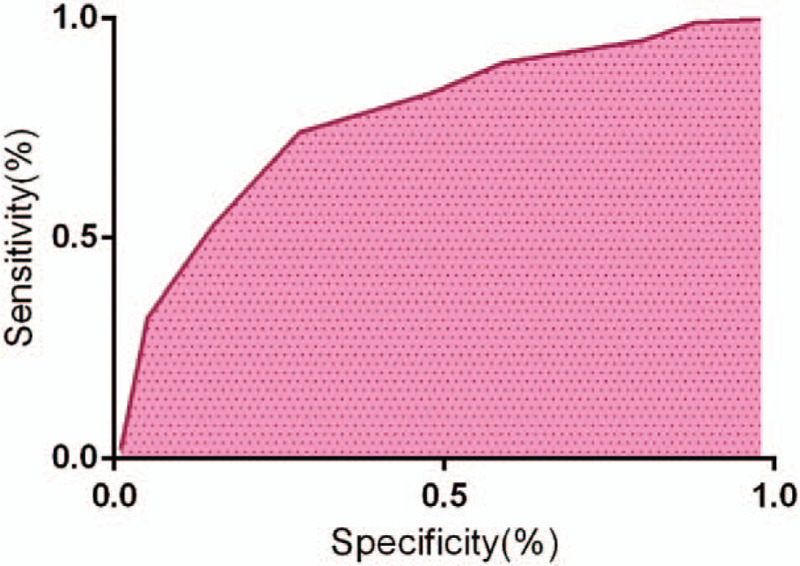
The receiver operating characteristic curve (ROC) curve of contrast-enhanced ultrasonography (CEUS) examination for preoperative sentinel lymph node (SLN) evaluation.

As Table 2 presents, a total of 248 SLNs were detected during the preoperative CEUS examination and the operation. The sensitivity of CEUS to the preoperative evaluation of SLN was 84.67%, the specificity was 81.14%, the positive predictive value was 76.08%, and the negative predictive value was 89.27%, the positive likelihood ratio was 4.06, and the negative likelihood ratio was 0.14. As Figure 1 presents, the area under the curve of the preoperative evaluation of SLN in CEUS examination was 0.813 (95% confidence interval: 0.765–0.911).

**Table 2:** The reference value of contrast-enhanced ultrasonography for sentinel lymph node detection.

Results	SLN detection	Pathological detection	χ^2^	*P*
SLN positive	36 (33.33%)	33 (30.56%)	1.205	1.184
SLN negative	72 (66.67%)	75 (69.44%)		

SLN = sentinel lymph node

### 3.3 The size distribution of detected SLN

**Figure 2:**
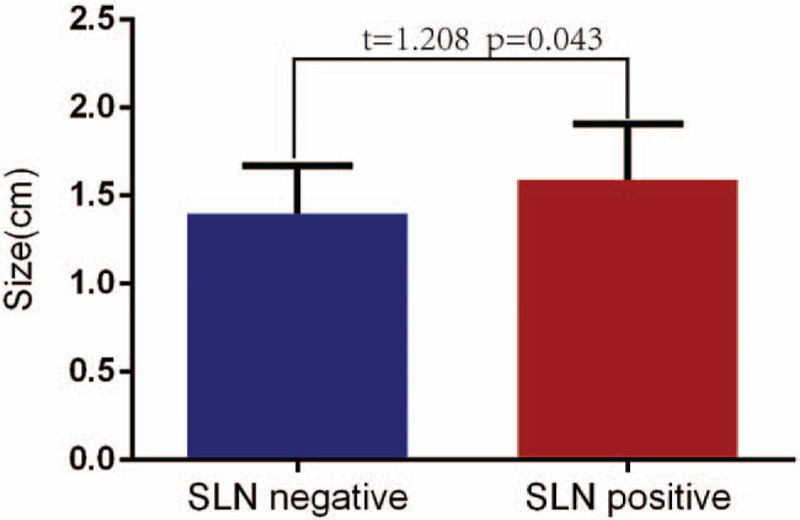
The size distribution of detected SLN. SLN = sentinel lymph node.

A total of 166 SLNs were detected in the SLN-negative group and 75 SLNs were detected in the SLN-positive group. As Figure 2 presents, there was significant difference in the size of SLNs between SLN-negative and SLN-positive groups (*P* = .043).

## 4 Discussion

SLN is the lymph node that breast cancer must pass through for lymph node metastasis.^[15]^ SLN detection can predict regional metastasis information to determine whether to perform regional lymph node dissection.^[16]^ The Chinese Anti-Cancer Association Guidelines and Standards for the Diagnosis and Treatment of Breast Cancer^[17]^ and the Clinical Practice Guidelines for Breast Cancer in the United States^[18]^ have clearly pointed out that SLN biopsy for breast cancer can accurately evaluate the pathological status of axillary lymph nodes and it is safe and effective for patients with negative axillary lymph nodes. It can replace ALND to significantly reduce the complications of surgery and improve the quality of life of patients.^[19]^

The principle of CEUS is to inject contrast agents through different paths to increase the contrast with the tissues and increase the display of tissues, organs, and lesions.^[20,21]^ At present, it has been widely used in clinical diagnosis of abdominal and superficial organ tumors, and differentiation of benign and malignant kidney tumors.^[22]^ CEUS examination provides a new idea for finding and predicting the presence or absence of metastasis of SLN.^[21,23,24]^ The results of this present study have found that CEUS can provide valuable information for SLN detection in patients with breast cancer, and CEUS has high clinical value for the location of SLN and prediction of metastasis before surgery.

CEUS has relatively high sensitivity and specificity for preoperative evaluation of SLN, but there are still some false negatives,^[25]^ which may be explained by following reasons. SLN metastasis is divided into macrometastasis and micrometastasis. For some patients with micrometastasis, no obvious anatomical changes have occurred in the lymph nodes, and it is difficult to find abnormalities in lymph node morphology and blood perfusion in contrast-enhanced ultrasound.^[26,27]^ When macrometastasis occurs in SLN, the lymphatic vessels are blocked, and the tracer cannot reach the SLN, but can pass through the bypass traffic branch to reach the lymph nodes that are not affected by cancer cells, so the first lymph node to be developed is mistaken for the SLN.^[28–30]^ it is the lymph nodes firstly be developed are not necessarily SLN in the true sense, resulting in missed diagnosis of CEUS.^[31]^ The SLN is obviously enhanced under acoustic contrast, and the image has good contrast.^[32]^ We can observe the running of the lymphatic vessels in real time and find the first enhanced lymph node.^[33]^ The whole process is clear and accurate. If necessary, the contrast agent microbubbles can be blasted again to observe the SLN. Preliminarily judging whether SLN transfers based on the performance of contrast-enhanced ultrasound imaging has advantages that other SLN detection methods that cannot match. It is worth noting that there are reports^[34–36]^ in the literature that contrast-enhanced ultrasound combined with methylene blue staining can achieve a success rate of 90.84% for SLN biopsy, a sensitivity of 95.28%, and a specificity of 100%. Therefore, CEUS may still need to be combined with other tracers to better identify the SLN.^[37]^

We did not find significant difference between SLN positive and negative in this study. It may be explained that size difference can be correlated to the metastasis stage of the tumor, and our sample size was small, it might be underpowered to detect the differences. Furthermore, we have found that there was a significant difference in the size of the SLN between the SLN-positive group and the negative group. The SLN of the positive group was significantly greater than that of the negative group. The size of SLN is an important factor that influences the diagnosis of SLN metastasis by contrast-enhanced ultrasound. Therefore, the SLN display images under clear contrast-enhanced ultrasound can be used to preliminarily determine whether SLN has metastasized. If metastasis is suspected, further treatment is required. In this way, there is no increase in trauma and overtreatment of the patient, and it is an objective indicator to help choose the surgical treatment plan.^[38]^

## 5 Conclusions

In conclusion, preoperative CEUS has certain predictive value for the presence of SLN in patients with breast cancer. CEUS can clearly show the detected lymphatic vessels and SLN, which is beneficial to guide SLN biopsy with accurate positioning, simple, and convenient advantages. It has broad prospects in clinical applications. Future studies are needed to further identify the role of CEUS in the early diagnosis of breast cancer.

## Author contributions

**Data curation:** Shihui Ma, Yuguang Xu.

**Formal analysis:** Shihui Ma, Feihai Ling.

**Investigation:** Shihui Ma, Yuguang Xu, Feihai Ling.

**Project administration:** Shihui Ma, Yuguang Xu, Feihai Ling.

**Resources:** Yuguang Xu, Feihai Ling.

**Software:** Yuguang Xu.

**Supervision:** Shihui Ma, Feihai Ling.

**Validation:** Shihui Ma, Yuguang Xu.

**Visualization:** Yuguang Xu.

**Writing – original draft:** Yuguang Xu, Feihai Ling.

**Writing – review & editing:** Feihai Ling.
